# Musical Sound Quality as a Function of the Number of Channels in Modern Cochlear Implant Recipients

**DOI:** 10.3389/fnins.2019.00999

**Published:** 2019-09-24

**Authors:** Katelyn Berg, Jack Noble, Benoit Dawant, Robert Dwyer, Robert Labadie, Virginia Richards, René Gifford

**Affiliations:** ^1^Department of Hearing and Speech Sciences, Vanderbilt University Medical Center, Nashville, TN, United States; ^2^Department of Electrical Engineering and Computer Science, Vanderbilt University, Nashville, TN, United States; ^3^Department of Otolaryngology, Vanderbilt University Medical Center, Nashville, TN, United States; ^4^Department of Cognitive Science, University of California, Irvine, Irvine, CA, United States

**Keywords:** cochlear implant, music, sound quality, channels, electrode placement

## Abstract

**Objectives:**

This study examined musical sound quality (SQ) in adult cochlear implant (CI) recipients. The study goals were to determine: the number of channels needed for high levels of musical SQ overall and by musical genre; the impact of device and patient factors on musical SQ ratings; and the relationship between musical SQ, speech recognition, and speech SQ to relate these findings to measures frequently used in clinical protocols.

**Methods:**

Twenty-one post-lingually deafened adult CI recipients participated in this study. Electrode placement, including scalar location, average electrode-to-modiolus distance ($\bar{M}$), and angular insertion depth were determined by CT imaging using validated CI position analysis algorithms (e.g., Noble et al., 2013; Zhao et al., 2018, 2019). CI programs were created using 4–22 electrodes with equal spatial distribution of active electrodes across the array. Speech recognition, speech SQ, music perception via a frequency discrimination task, and musical SQ were acutely assessed for all electrode conditions. Musical SQ was assessed using pre-selected musical excerpts from a variety of musical genres.

**Results:**

CI recipients demonstrated continuous improvement in qualitative judgments of musical SQ with up to 10 active electrodes. Participants with straight electrodes placed in scala tympani (ST) and pre-curved electrodes with higher $\bar{M}$ variance reported higher levels of musical SQ; however, this relationship is believed to be driven by levels of musical experience as well as the potential for preoperative bias in device selection. Participants reported significant increases in musical SQ beyond four channels for all musical genres examined in the current study except for Hip Hop/Rap. After musical experience outliers were removed, there was no relationship between musical experience or frequency discrimination ability and musical SQ ratings. There was a weak, but significant correlation between qualitative ratings for speech stimuli presented in quiet and in noise and musical SQ.

**Conclusion:**

Modern CI recipients may need more channels for musical SQ than even required for asymptotic speech recognition or speech SQ. These findings may be used to provide clinical guidance for personalized expectations management of music appreciation depending on individual device and patient factors.

## Introduction

The cochlear implant (CI) is the most successful sensory prosthetic device to date, yielding significant improvements in speech understanding (e.g., Holden et al., 2013) and quality of life (e.g., McRackan et al., 2017) for the majority of recipients. Despite its success for restoration of auditory detection and speech recognition, music perception and appreciation remain major challenges for most CI recipients, due to a number of factors including poor pitch and timbre perception as well as reduced spectral resolution (e.g., Kang et al., 2009; Jung et al., 2012) as well as potential for poor auditory neural health. Focusing on the latter, the classic literature concluded that CI recipients have limited access to spectral cues due to channel interaction (spread of electrical excitation). A discrete number of 5–10 independent channels may be available to these recipients for various speech and auditory measures, despite having up to 12–22 intracochlear electrodes (Fishman et al., 1997; Friesen et al., 2001, 2005). These previous studies were completed using older-generation speech coding strategies with patients implanted using more traumatic surgical approaches, unknown electrode placement, and stricter candidacy criteria. Patients in these earlier studies also had less residual hearing, poorer speech understanding, and longer durations of deafness compared to modern CI recipients (e.g., Holder et al., 2018b). Several factors limit the precision of intracochlear electrical stimulation and negatively affect spectral resolution, including: (1) channel interaction, which has been shown to span one-third or more of the array (e.g., Hughes et al., 2013; Padilla and Landsberger, 2016); (2) the amount of viable spiral ganglion cells along the length of the cochlear duct, which is currently unable to be quantified; and (3) electrode placement within the cochlea, which is unknown for the majority of patients due to a lack of postoperative imaging. Electrode placement is especially critical as multiple studies have documented that the electrode–neural interface is rarely uniform along the array with distances ranging from 0 to 2 mm from the closest modiolar location (Davis et al., 2016) and 13% of implanted devices have extracochlear electrodes not referenced in the operative report (Holder et al., 2018a).

More recent studies have re-examined speech recognition and spectral resolution abilities for CI recipients implanted under current expanded criteria, using atraumatic electrode design and surgical approaches as well as speech coding strategies used in today’s clinic. These latest reports suggest that modern-day CI recipients with pre-curved electrode arrays may have greater channel independence than previous generation recipients. Croghan et al. (2017) investigated channel independence for newer generation adult CI recipients with Nucleus devices. For sentence recognition at various signal-to-noise ratios (SNRs), they reported better performance using 22 active electrodes with 8 maxima versus 12 active electrodes with 8 maxima. However, they kept the number of maxima constant in an n-of-m strategy irrespective of the number of active electrodes. Another limitation of the Croghan et al.’s (2017) study was the lack of image-based confirmation of electrode location for the nine pre-curved electrode recipients: an electrode documented to result in translocation (ST–SV) in up to 42% of cases (Wanna et al., 2014). Berg et al. (2019) investigated channel independence for a group of 11 adult recipients with Nucleus pre-curved arrays, verified by postoperative imaging to be completely in the scala tympani (ST). They reported significant increases in speech recognition in noise with increasing channels up to 22 channels with 16 maxima (16-of-22), compared to continuous interleaved sampling (CIS) maps with 4–10 channels; considering CIS alone, 16-channel CIS provided significantly higher speech recognition in quiet than even 10-channel CIS. Berg et al. (2019) also reported significantly higher speech recognition in quiet and noise with 16 maxima (16-of-22) compared to 8 maxima (8-of-22) – an effect significantly correlated with mean electrode-to-modiolus distance along the implanted array. Because lower electrode-to-modiolus distances – associated with well-placed pre-curved electrodes that evenly hug the modiolar wall – require less charge for upper stimulation levels (e.g., Davis et al., 2016), CI recipients with pre-curved electrodes in ST may experience less channel interaction (e.g., Chatterjee and Shannon, 1998), affording better spectral resolution. Given recent evidence for greater channel independence for adult CI recipients in the speech domain, the current study wanted to investigate the effect of channels and channel independence on the largely unexplored music domain.

Poor spectral resolution has also contributed to poor pitch discrimination, as well as melody and timbre identification for CI recipients (e.g., McDermott, 2004; Moore and Carlyon, 2005; Drennan and Rubinstein, 2008; Won et al., 2010). Nimmons et al. (2008) found that nearly all of their participants had F0 discrimination ability between 1 and 6 semitones, but varied significantly more with complex-tone pitch discrimination, ranging from less than 1 semitone up to 12 semitones. However, few studies have focused on assessing musical SQ with CIs, even though CI recipients report significant musical SQ impairments following implantation, and musical SQ is rated as the most significant factor responsible for music listening enjoyment (Lassaletta et al., 2008a, b; Roy et al., 2012). This is problematic because no clear relationship exists between music perceptual accuracy and the perceived SQ of music (Gfeller et al., 2008; Looi et al., 2011). While music perception or musical SQ are rarely assessed in the Audiology clinic, speech recognition and speech SQ are addressed through regular evaluations and programming adjustments. It is unknown if a relationship exists between speech recognition performance and SQ, and musical SQ; and if speech measures can suffice for addressing musical SQ impairments.

The purpose of this study was to assess musical SQ in adult CI recipients implanted under expanded criteria and current technology to determine: (a) the number of channels needed to achieve the highest level of overall musical SQ; (b) the impact of device factors, such as electrode type, electrode scalar location, mean electrode-to-modiolus distance, variance in electrode-to-modiolus distance, insertion depth of the electrode array, surgeon, and implant manufacturer on these ratings; (c) the impact of musical genre on the number of channels needed to achieve asymptotic ratings of musical SQ; (d) the impact of patient factors, such as musical experience, music perception abilities via a frequency discrimination task, duration of CI use (since activation), and pre-operative hearing thresholds; and (e) the relationship between musical SQ, speech recognition, and speech SQ to understand how these findings relate to measures frequently used in clinical protocols.

Given that music is spectrally complex, we hypothesized that lower electrode-to-modiolus distance would lead to increased ratings of musical SQ due to less channel interaction. While older studies were limited by the existing technology, modern advances in electrode design, surgical technique, candidacy criteria, and imaging have increased the potential for greater channel independence (e.g., Croghan et al., 2017; Berg et al., 2019). Due to these advances, the current study was able to assess subjective musical SQ over a wider range of participants and devices than previous work. Specifically, our hypotheses were that: (1) musical SQ ratings would continue to increase with more available independent channels; (2) pre-curved electrodes positioned in the ST would show increased ratings in musical SQ compared to straight or translocated (ST–SV) pre-curved and straight electrodes; (3) participants with more musical experience, better frequency discrimination ability, and longer device use would rate musical SQ higher than participants with less experience and ability; and (4) CI recipients with better speech recognition performance and speech SQ ratings would also rate musical SQ as better than poorer-performing CI recipients.

## Materials and Methods

### Study Participants

**FIGURE 1:**
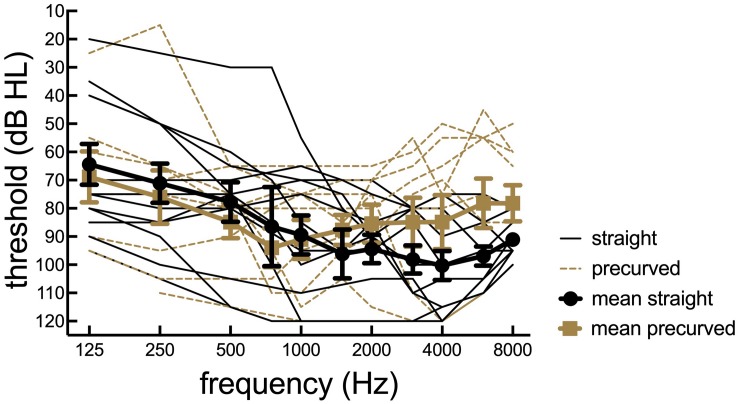
Participant preoperative hearing thresholds. Individual thresholds (dB HL) as a function of pure tone frequency (Hz) for 21 cochlear implant listeners. Straight electrode recipients are plotted in dotted lines and pre-curved recipients are plotted in solid black lines.

Twenty-one postlingually deafened adult CI recipients (range = 34–80 years; mean age = 58.8 ± 13.5 years) participated. Each of the three FDA-approved CI manufacturers were represented with seven Advanced Bionics (AB), seven Cochlear, and seven MED-EL recipients. Of the participants with AB devices, there was one 1J recipient, three Mid-Scala, and three SlimJ electrodes. Of the participants with cochlear devices, there were three CI512, two CI532, and two CI522 electrodes. Of the Med-El participants, there was one Standard and six Flex28 electrodes. Surgeries were performed by five different surgeons at the authors’ current institution and four surgeons at outside institutions. The type of surgical approach (i.e., cochleostomy, round window, extended round window) was not reported or available for all of our participants, so this was not included in the current study. Of the 21 participants, 5 were unilateral CI recipients without contralateral amplification, 8 were bimodal CI recipients indicating a CI on the tested ear and a contralateral hearing aid, 5 were bilateral CI recipients, and 3 used electric-acoustic stimulation (EAS) and a contralateral hearing aid. See Figure 1 for participant pre-operative hearing thresholds. Participants using EAS in their clinical map were converted to full bandwidth programs and acoustic stimulation was deactivated. All testing was completed in the CI-alone condition. Participants with residual hearing in the contralateral ear were occluded using an E.A.R plug in addition to a circum-aural ear muff. Inclusion criteria required at least 6 months of CI experience and at least 14, 18, and 10 active electrodes in use for AB, Cochlear, and MED-EL, respectively. Participants also needed to score at least 20% correct on AzBio sentences in +5 dB SNR with their clinical map to avoid floor effects. Table 1 provides demographic information.

**TABLE 1:** Demographic information.

**ID**	**Implant manufacturer**	**Channel stimulation rate**	**Number of active channels**	**Electrode type**	**Electrode**	**Ear implanted**	**Scalar location**	**Insertion depth**	**$\bar{M}$**	**CI experience (months)**	**Frequency discriminatory. threshold (semitones)**	**Ollen index**	**LF Pre-op PTA (0.25, 0.5, 0.75 kHz)**	**Pre-op PTA (0.5, 1, 2, 4 kHz)**
S1	AB	1547	16	Pre-curved	Mid-Scala	R	ST	354.28	0.51	46	1.5	18	78.3	106.3
S2	MED-EL	1207	12	Straight	Standard	L	ST	446.83	1.26	29	1.4	118	102.5	110
S3	Cochlear	900	20(1–2)	Pre-curved	CI512	R	ST	356.09	0.44	12	0.4	218	105	86.3
S4	AB	2855	15(16)	Pre-curved	Mid-Scala	R	ST	392.41	0.53	11	1.4	31	88.3	90
S5	Cochlear	900	21(1)	Pre-curved	CI512	R	ST–SV	396.56	0.59	23	0.5	87	40	73.8
S6	MED-EL	1237	12	Straight	Flex28	R	ST	499.72	1.12	46	0.4	273	78.3	85
S7	MED-EL	1247	11(12)	Straight	Flex28	R	ST–SV	466.56	1.47	34	0.5	18	80	73.8
S8	AB	3535	14(15–16)	Pre-curved	Mid-Scala	R	ST	439.98	0.58	26	0.4	261	73.3	70
S9	MED-EL	1389	12	Straight	Flex28	R	ST	509.82	1.37	34	1.7	102	80	92.5
S10	MED-EL	1207	11(2)	Straight	Flex28	L	ST	406.38	1.30	67	1.4	104	57.5	82.5
S11	AB	1547	15(16)	Pre-curved	Mid-Scala	R	ST	326.23	0.59	23	0.4	162	77.5	76.3
S12	MED-EL	1210	11(12)	Straight	Flex28	R	ST	305.85	1.24	57	3.5	33	82.5	88.8
S13	MED-EL	1207	12	Straight	Flex28	R	ST	584.04	1.18	70	1.8	132	70	75
S14	AB	3712	15(16)	Straight	SlimJ	R	ST	508.34	1.13	13	0.4	304	75	107.5
S15	Cochlear	900	22	Pre-curved	CI512	L	ST–SV	375.21	0.59	129	1.8	71	110	118.8
S16	AB	3712	14(15–16)	Straight	SlimJ	R	ST	339.87	1.17	11	0.4	779	28.3	68.8
S17	Cochlear	900	21(1)	Straight	CI522	L	ST	481.26	1.09	23	6.5	275	108.3	118.8
S18	Cochlear	900	20(1–2)	Pre-curved	CI532	L	ST	401.75	0.44	29	0.5	31	67.5	61.3
S19	Cochlear	900	19(1–3)	Pre-curved	CI532	L	ST	430.49	0.39	19	0.8	98	88.3	87.5
S20	AB	2184	14(9,16)	Straight	1J	L	ST-SV	322.47	1.18	187	0.6	94	113.3	118.8
S21	Cochlear	900	22	Straight	CI522	R	ST	351.61	0.96	18	0.5	719	60	76.3
Mean										43.2	1.3	187.1	79.2	88.9
*SD*										42.9	1.4	207.5	21.9	17.8

*Channel stimulation rate was set from the clinical map and kept constant throughout all conditions. Number of active channels in the clinical map are listed; the clinically deactived channels are listed in parentheses. Insertion depth is represented in degrees. Average electrode to modiolus distance ($\bar{M}$) is represented in millimeters. Cochlear implant experience is represented in months since date of activation for tested ear Hearing thresholds displayed in dB HL.*

### Conditions and Materials

All experimental activities were completed in accordance with IRB approved protocols at the Vanderbilt University and the Vanderbilt University Medical Center. Electrode placement, including scalar location, mean and variance of electrode-to-modiolus distance, and angular insertion depth were all determined by CT imaging using validated CI position analysis algorithms (Noble et al., 2013; Zhao et al., 2018, 2019). These algorithms were created using a statistical shape model of 10 cadaver temporal bone microCT images. The statistical shape model was then built onto each participant’s pre-operative clinical CT scan to determine the scala divisions within the cochlea. The participant’s post-implantation CT scan was then fit onto their pre-operative CT to enable calculating the exact location of each individual electrode with respect to scalar location and the distance to the nearest modiolar surface. The average electrode to modiolus distance ($\bar{M}$) and variance across the arrays were then calculated from these measurements.

Cochlear implant programs were created for electrode counts ranging from 4 to 22 with equal spatial distribution of active electrodes across the array to follow the electrode deactivation methods of Friesen et al. (2001). For all conditions, the frequency map was automatically re-allocated based on the number of active electrodes to simulate a clinical manipulation. It is possible that SQ ratings were affected by these acute manipulations due to the participant’s lack of experience listening to music with the maps used in this study. Refer to Table 2 for specific electrodes activated to achieve the spatially selective maps. Note the bandwidths of the 16-channel map and the clinical map for AB participants differed slightly due to the difference in stimulation type (CIS versus current steering strategies). For participants with electrodes deactivated clinically, the adjacent electrode was activated if the electrode condition required an electrode to be active that had been clinically deactivated. All experimental programs used a classic CIS (Wilson et al., 1991) stimulation strategy except for the participants’ clinical maps. The participants’ clinical maps all used iterations of CIS including Optima-S, Advanced Combination Encoder (or n-of-m), and FS4 for AB, Cochlear, and MED-EL, respectively. All of the clinical maps used the highest number of active electrodes possible for that participant (Table 1).

**TABLE 2:** Active electrodes by condition and manufacturer.

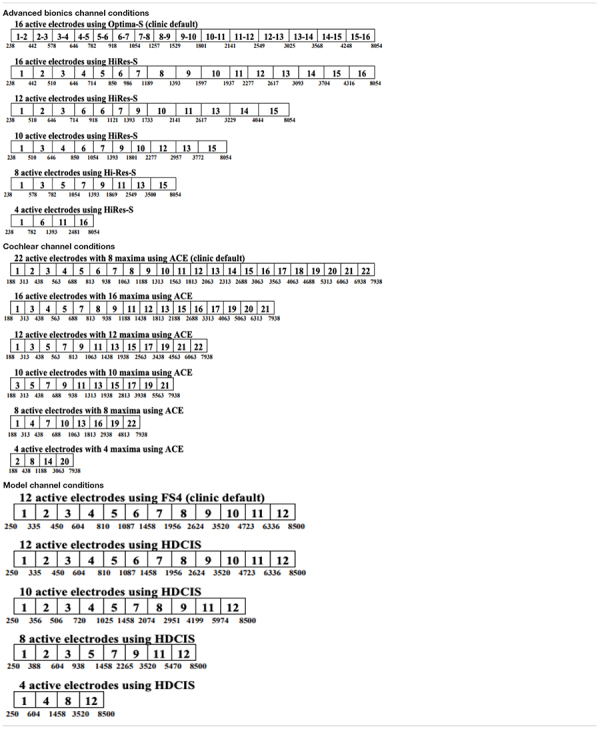

*Frequency tables for all channel conditions tested each cochlear implant manufacturer. Large numbers within cells indicate the electrode number that is active for that condition. Small numbers underneath cells indicate bandwidths of channels in Hertz (Hz).*

Channel stimulation rate and pulse duration were kept constant across all conditions, but manufacturer dependent (Table 1). Threshold levels were not adjusted from the participant’s own map; however, aided detection thresholds were verified to be within 15–30 dB HL from 250 to 6000 Hz before the participant began the study. Upper stimulation levels were globally adjusted using the participants’ own maps to achieve equivalent loudness across all experimental maps. All front-end processing features were deactivated, with the exception of Autosensitivity Control (ASC) and Adaptive Dynamic Range Optimization (ADRO) for cochlear participants as all participants were longtime users of ASC and ADRO.

Electrode condition and measure assessment order were both randomized using a Latin Square design. All testing was completed acutely. Each of the conditions was tested using a loudspeaker at 0-degrees azimuth and 1 m from the participant in a single walled sound booth using: CNC monosyllabic words (Peterson and Lehiste, 1962) and AzBio sentences (Spahr et al., 2012) in +5 SNR using 20-talker babble noise. One list of CNC words and AzBio sentences in +5 dB SNR was presented for each channel condition; lists were only used once per participant. Target speech stimuli were presented at a calibrated level of 60 dB SPL. Subjective SQ judgments were assessed using a visually presented 10-point scale (1 = very poor; 10 = very good), in which the participant rated the overall SQ of the list of CNC words and AzBio sentences in +5 dB SNR for each condition. Prior to statistical analyses, all speech recognition scores were converted to rationalized arcsine units (RAUs) (Studebaker, 1985) and all speech SQ ratings were converted to *z*-scores.

Musical SQ was assessed using a randomly selected subset of 15 30-s song clips from a group of 30 possible songs, all from various genres and styles. The clips were presented at a comfortable listening level, kept constant for all conditions, and the participant was asked to make subjective SQ judgments immediately after they listened to the clip. Participants were given verbal instructions prior to beginning the musical SQ task, asking them to select their rating for each musical excerpt based on the clarity, richness, and pleasantness of the voices and instruments and not how much they liked or were familiar with the excerpt. The participant typed in their rating on a keypad after each clip using a 10-point scale (0 = very poor; 9 = very good) presented on a touch-screen computer. Prior to analyses, all of the musical SQ ratings were converted to *z*-scores. The transformed *z*-scores for mean overall musical SQ ratings of all 15 clips for each condition as well as the mean musical SQ rating for each genre for each condition were used for analyses. The musical genres were determined by the record label’s description of each song. The musical genres used for analyses included Alternative, Hip Hop and Rap, Jazz, Popular, Rhythm and Blues (R&B), and Rock n Roll. Within each condition, an individual participant could potentially listen to up to six songs from the same genre. Participants listened to at least one sample from each genre for every condition.

A measure of frequency discrimination was also assessed for each condition via the frequency discrimination test in Angel Sound^1^. An adaptive, three-alternative forced-choice (3AFC) procedure was used to determine the frequency change threshold. In each trial, the participant would listen to a series of three pure tones, two reference tones (440 Hz), and the target tone. The target tone varied in the number of semitones it differed from the reference tone, always ascending. The order of reference and target stimuli was randomized. The participant was asked to select the target (which one is different from the other two tones) by tapping one of three boxes on a touchscreen computer and without feedback. The step size adjusted according to a two-down one-up staircase technique based on the participants’ response. The transformed up-down staircase technique was used to track the 79% correct point on the psychometric function. Important to note, a score of less than a 0.5 semitone threshold was not possible due to the set-up of the task (Zhang et al., 2019).

Participants also completed the Ollen Musical Sophistication Index (OMSI) as part of the study to help classify them as more or less musically sophisticated (Ollen, 2006). Musical sophistication includes the participant’s knowledge about music; her ability to play a musical instrument or sing; and to understand, respond to, and create music. The OMSI is a 10-item questionnaire that yields a numerical score indicating the probability (in percent times 10) that a music expert would categorize the participant as “more musically sophisticated.” Participants with scores greater than 500 are considered “more musically sophisticated,” while participants with scores less than 500 are considered “less musically sophisticated.” See Table 1 for individual participant Ollen scores.

## Results

### Number of Channels Needed for Musical Sound Quality and Impact of Device Factors

**FIGURE 2:**
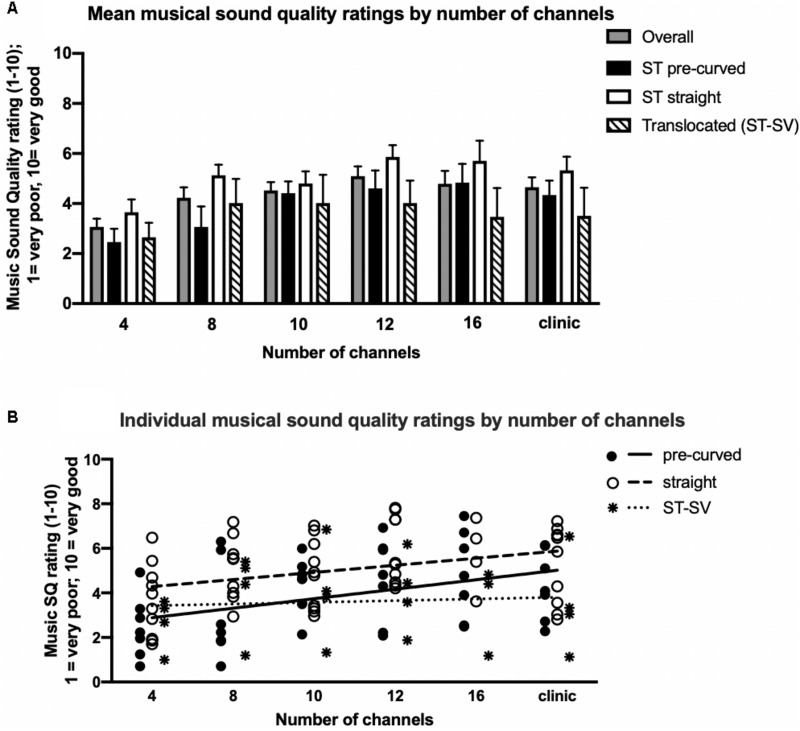
Musical sound quality ratings as a function of the number of channels by electrode type and scalar location. Mean **(A)** and individual **(B)** musical sound quality ratings for 21 cochlear implant listeners across all tested channel conditions. In panel **A**, mean data for all listeners combined (solid gray), ST pre-curved (solid black), ST straight (solid white), and ST-SV electrode recipients (diagonal black stripes) are shown. Error bars are +1 SEM. In panel **B**, individual data for ST pre-curved electrode recipients (solid black circles) with the group mean shown (solid line), ST straight electrode recipients (solid white circles) with the group mean shown (dashed line), and ST–SV electrode recipients (black stars) with the group mean shown (dotted line).

A linear mixed model was completed with the number of channels, scalar location, and electrode type as independent variables and musical SQ ratings as the dependent variable. *Post hoc* analyses were completed using a Sidak adjustment with all-pairwise, multiple comparisons. For overall musical SQ ratings, there was a significant main effect of number of channels [*F*_(__5__,__31__)_ = 5.007, *p* = 0.002], electrode type [*F*_(__1__,__108__)_ = 17.363, *p* < 0.001], and electrode scalar location [*F*_(__1__,__108__)_ = 5.747, *p* = 0.018]. There was no significant interaction between electrode type and scalar location for this sample [*F*_(__1__,__109__)_ = 2.286, *p* = 0.133]. The raw data for these comparisons are displayed in Figure 2 with panels A and B displaying mean and individual data, respectively. *Post hoc* analyses revealed significant performance differences between 4 and 10 channels (*p* = 0.035), 4 and 12 channels (*p* = 0.001), 4 and 16 channels (*p* = 0.026), and 4 channels and the clinic map (*p* = 0.023). No other channel comparisons were statistically significant for this sample.

Straight electrode recipients (mean = 4.727, *SD* = 0.234) demonstrated significantly higher overall musical SQ ratings compared to pre-curved electrode recipients (mean = 3.543, *SD* = 0.237, *t*_19_ = 14.53, *p* < 0.001), though on average, all recipients reported generally neutral to poor musical SQ ratings. Participants with electrodes completely in the ST (mean = 4.567, *SD* = 0.165) demonstrated significantly higher overall musical SQ ratings compared to patients with translocated electrodes (mean = 3.703, *SD* = 0.328, *p* = 0.019). Figure 2B displays linear regression fits for the pre-curved (solid line), straight (dashed line), and translocated (dotted line) electrode recipients. Regression analysis revealed that the regression slope coefficient was significantly different from zero for pre-curved recipients only [*F*_(__1__,__4__)_ = 9.0, *p* = 0.04]. For both straight electrode [*F*_(__1__,__4__)_ = 5.1, *p* = 0.09] and translocated electrode recipients [*F*_(__1__,__4__)_ = 0.29, *p* = 0.58], the regression slope coefficient was not significantly different from zero.

The effect of manufacturer on overall musical SQ ratings was examined using a one-way ANOVA was completed with CI manufacturer as the independent variable and overall musical SQ ratings using the clinical map was the dependent variable. There was no significant effect of manufacturer on overall musical SQ ratings of this sample [*F*_(__2__,__18__)_ = 0.78, *p* = 0.473]. The effect of surgeon on overall musical SQ ratings was also examined using a one-way ANOVA with surgeon as the independent variable and overall musical SQ rating using the clinical map as the dependent variable. There was no significant effect of surgeon for this sample [*F*_(__5__,__15__)_ = 1.493, *p* = 0.250].

**FIGURE 3:**
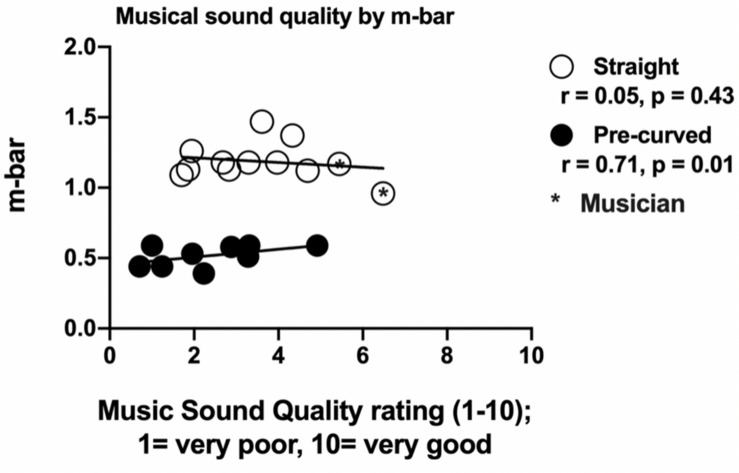
Impact of average electrode to modiolus distance ($\bar{M}$) and variance of $\bar{M}$ on musical sound quality ratings with clinical map by electrode type. Individual average electrode to modiolus distance ($\bar{M}$) values and variance of $\bar{M}$ across the array (*y*-axis) as a function of musical sound quality ratings using the participant’s clinical map (*x*-axis). Black circles represent pre-curved electrodes and white circles represent straight electrodes. Of note, variability of $\bar{M}$ for the pre-curved electrode sample is smaller than the general clinical population. Of note, the two white circles farthest to the right are the two musicians.

Pearson correlations were used to examine the relationship between overall musical SQ ratings using the clinical map and average electrode-to-modiolus distance, the variance in electrode-to-modiolus distance across the array, and electrode insertion depth in degrees. There was a significant positive correlation between mean electrode-to-modiolus distance ($\bar{M}$, in mm) and overall musical SQ ratings (*r* = 0.28, *p* = 0.002), meaning higher $\bar{M}$ was associated with better musical SQ ratings. $\bar{M}$ was further examined by electrode type and a significant positive correlation was found between $\bar{M}$ of pre-curved electrodes and overall musical SQ ratings (*r* = 0.71, *p* = 0.01). $\bar{M}$ as a function of overall musical SQ ratings (raw data) is displayed in Figure 3. There was no significant relationship between $\bar{M}$ of straight electrodes and overall musical SQ ratings (*r* = 0.05, *p* = 0.43), likely due to the relative homogeneity of $\bar{M}$ values for the straight electrode recipients (Table 1). To better understand why a higher $\bar{M}$ value would lead to higher overall musical SQ ratings, the variance in electrode-to-modiolus distances across the array was also examined. There was a positive significant relationship between greater $\bar{M}$ variance and higher musical SQ ratings for those electrodes completely in ST (*r* = 0.51, *p* = 0.03), but not for those in ST–SV (*r* = 0.77, *p* = 0.22). By electrode type, there was a positive significant relationship between greater $\bar{M}$ variance and higher musical SQ ratings for pre-curved electrodes (*r* = 0.95, *p* = 0.001), but not for straight electrodes (*r* = 0.25, *p* = 0.47). There was no significant relationship between insertion depth of the electrode measured in degrees and overall musical SQ ratings (*r* = 0.03, *p* = 0.768).

### Impact of Musical Genre on Number of Channels Needed for Musical Sound Quality Ratings

**FIGURE 4:**
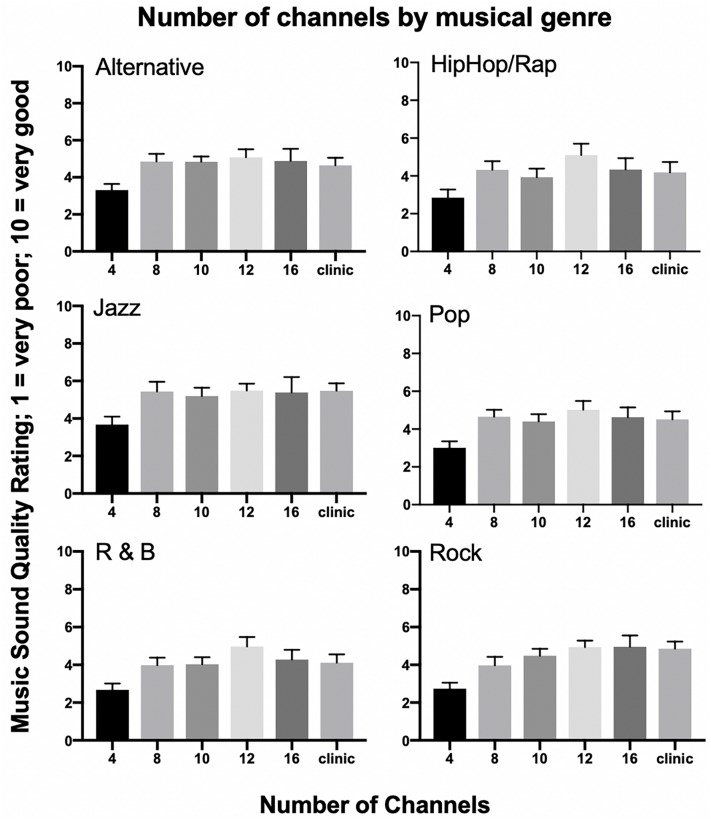
Number of channels by musical genre. Mean musical sound quality ratings for six musical genres, including Alternative, Hip Hop/Rap, Jazz, Pop, Rhythm and Blues (R&B), and Rock for 21 cochlear implant listeners across all tested channel conditions. Error bars are +1 SEM.

The effect of musical genre on the number of channels as a function of musical SQ was examined using a linear mixed model with number of channels as the independent variable and musical SQ ratings by genre (Alternative, Hip Hop and Rap, Jazz, Popular, R&Bs, and Rock n Roll) as the dependent variable. Figure 4 shows this analysis using the raw data for musical SQ ratings. *Post hoc* analyses included paired *t*-tests between channel conditions for each musical genre. For Alternative music, there was a significant main effect of number of channels [*F*_(__5__,__30__)_ = 3.38, *p* = 0.016]. *Post hoc* analyses revealed significant performance differences between 4 and 10 channels (*t* = −4.594, *p* < 0.001) and 4 and 12 channels (*t* = −5.692, *p* < 0.001). None of the other channel comparisons were significantly different for alternative music. For Hip Hop and Rap, there was no significant main effect of number of channels [*F*_(__5__,__36__)_ = 2.292, *p* = 0.06]. For Jazz, there was a significant main effect of number of channels [*F*_(__5__,__30__)_ = 2.676, *p* = 0.041]. *Post hoc* analyses for Jazz revealed significant performance differences between 4 and 12 channels (*t* = −4.893, *p* < 0.001). None of the other channel comparisons were significantly different for jazz music SQ ratings. For Popular music, there was a significant main effect of number of channels [*F*_(__5__,__36__)_ = 3.592, *p* = 0.010]. *Post hoc* analyses revealed significant performance differences between 4 and 8 channels (*t* = −4.478, *p* < 0.001) and 4 and 12 channels (*t* = −4.972, *p* < 0.001). None of the other channel comparisons were significantly different for popular music. For R&Bs, there was a significant main effect of number of channels [*F*_(__5__,__38__)_ = 3.744, *p* = 0.007]. *Post hoc* analyses revealed significant performance differences between 4 and 12 channels (*t* = −4.123, *p* = 0.001). None of the other channel comparisons were significantly different for R&B. For Rock n Roll, there was a significant main effect of number of channels [*F*_(__5__,__33__)_ = 6.229, *p* < 0.001]. *Post hoc* analyses revealed significant performance differences between 4 and 10 channels (*t* = −4.468, *p* < 0.001), 4 and 12 channels (*t* = −5.317, *p* < 0.001), as well as 4 and the clinical map with all active electrodes (*t* = −4.798, *p* < 0.001). None of the other channel comparisons were significantly different for Rock n Roll.

### Impact of Patient Factors on Musical Sound Quality

**FIGURE 5:**
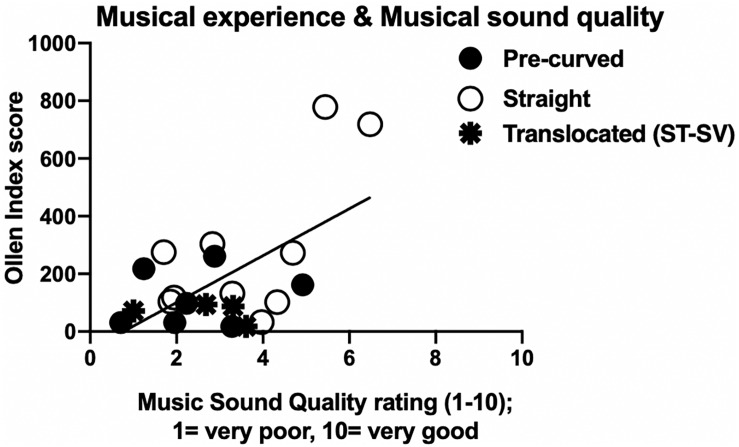
Impact of patient musical experience on musical sound quality ratings with clinical map. Individual Ollen Index of Musical Sophistication scores (*y*-axis) as a function of musical sound quality ratings using the clinical map (*x*-axis). Higher Ollen Index scores indicate more musical experience. Black circles represent pre-curved electrodes and white circles represent straight electrodes. Translocated electrodes that include two pre-curved and two straight electrode recipients are represented by the black stars.

Pearson correlations were used to examine the relationship between musical experience as measured by the Ollen index, pre-operative hearing thresholds, measured at the CI work-up appointment, music perception ability via frequency discrimination thresholds, and CI experience, in months, since activation. See Table 1 for Ollen scores, pre-operative pure tone averages, frequency discrimination thresholds, and duration of CI experience by participant. Musical SQ ratings with the clinical map were used for these analyses. There was a significant positive correlation between Ollen index of Musical Sophistication scores and overall musical SQ ratings (*r* = 0.40, *t* = −10.086, *p* < 0.001); that is, individuals with more musical experience rated musical SQ higher than individuals with less musical experience, as shown in Figure 5 using the raw data for musical SQ ratings. When the impact of musical experience is further broken down by electrode type, there is a significant positive correlation between musical experience and musical SQ ratings for straight electrodes (*r* = 0.67, *p* = 0.03), but there was no significant relationship between musical experience and musical SQ ratings for pre-curved electrodes (*r* = 0.19, *p* = 0.67). However, when the two outliers with greater Ollen scores are removed, the positive correlation is no longer significant (*r* = 0.11, *p* = 0.80). The relationship between pre-operative hearing thresholds, using both a low-frequency pure tone average (LFPTA) (250,500, and 750 Hz) and a standard four frequency pure tone average (500, 1000, 2000, and 4000 Hz), and overall musical SQ ratings was examined in an attempt to understand why straight electrode recipients rated musical SQ better than pre-curved recipients. Pre-operative hearing thresholds are shown in Figure 1. However, there was no significant relationship between overall musical SQ ratings and LFPTA (*r* = 0.22, *p* = 0.35) or using the standard pure tone average (*r* = 0.06, *p* = 0.79). There was also no significant relationship between music perception ability measured via the AngelSound frequency discrimination task (*r* = −0.23, *p* = 0.50) or CI experience and overall musical SQ ratings (*r* = −0.116, *p* = 0.21).

### Relationship Between Musical Sound Quality, Speech Recognition, and Speech Sound Quality

**FIGURE 6:**
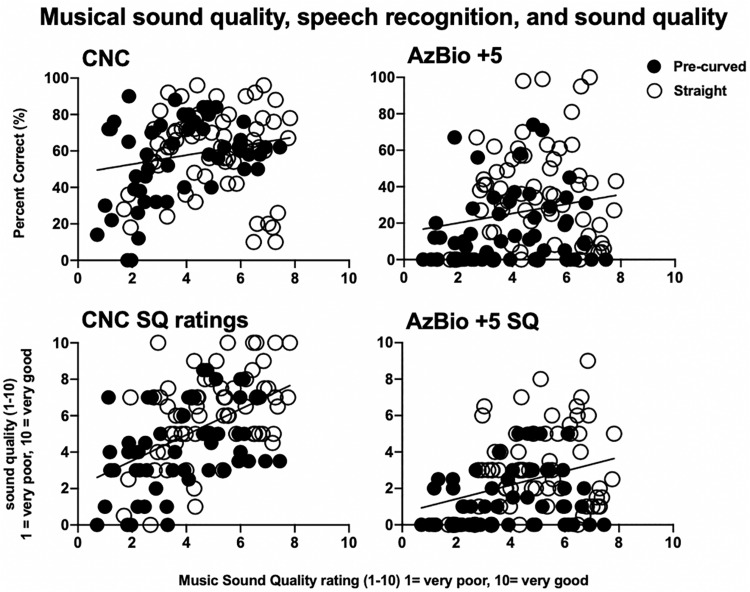
Musical sound quality ratings compared to speech recognition scores and sound quality ratings. Individual speech recognition for CNC monosyllabic words in quiet and AzBio sentences at +5 dB SNR **(top row)** in percent correct and speech sound quality for CNC monosyllabic words in quiet and AzBio sentences at +5 dB SNR **(bottom row)** as a function of musical sound quality ratings (*x*-axes). Black circles represent pre-curved electrodes and white circles represent straight electrodes.

Pearson correlation analyses were completed for *z*-transformed scores of overall musical SQ ratings and speech recognition scores in RAU (CNC words and AzBio sentences at +5 dB), as well as speech SQ ratings. However, for easier translation to the Audiology clinic, Figure 6 displays speech recognition scores in percent correct along with the raw SQ ratings. Musical SQ ratings using the clinical map were used for these analyses. For measures of speech recognition, there was a significant, but weak positive correlation between CNC word recognition in RAU and overall musical SQ ratings (*r* = 0.20, *t* = −27.636, *p* = 0.027). Similarly, there was a significant, but weak positive correlation between AzBio sentence recognition in noise in RAU and overall musical SQ ratings (*r* = 0.22, *t* = −7.268, *p* = 0.017). For transformed *z*-scores of speech SQ, there was a significant positive correlation between CNC SQ ratings and overall musical SQ ratings (*r* = 0.51, *t* = −26.492, *p* < 0.001), as well as a significant positive correlation between SQ ratings for AzBio in noise and overall musical SQ ratings (*r* = 0.33, *t* = −12.622, *p* < 0.001).

## Discussion

Hypothesis 1: *Musical SQ ratings will continue to increase with more available independent channels for pre-curved electrodes positioned in ST compared to straight or translocated (ST–SV) electrodes.*

Consistent with our primary hypothesis, adult CI recipients demonstrated continuous gains in musical SQ ratings with 10–22 channels compared with four channels. Regression analysis revealed that the increases in music SQ with number of channels was significant for pre-curved electrode recipients, but not for straight or translocated electrode recipients (Figure 2B). This finding is consistent with recent studies evaluating the number of channels needed for speech recognition with modern CI recipients (e.g., Croghan et al., 2017; Berg et al., 2019) suggesting that a greater number of independent channels may be both available and necessary than previously thought for asymptotic speech recognition and musical SQ, particularly for pre-curved electrode recipients. Also consistent with our primary hypothesis, participants with electrodes completely in ST reported significantly higher overall ratings for musical SQ than those with translocated arrays, emphasizing the importance of electrode insertion and resultant scalar location. Of note, this sample did have lower translocation rates for pre-curved electrodes (2/9 = 22%) and slightly higher translocation rates for straight electrodes (2/12 = 16.7%) than is reported in the literature (e.g., Wanna et al., 2014).

Contrary to our primary hypothesis and recent evidence, however, straight electrode recipients reported higher overall musical SQ ratings than pre-curved electrode recipients. In fact, the strong positive correlation between mean electrode-to-modiolus distance ($\bar{M}$) and overall musical SQ ratings suggest that arrays farther away from the modiolus (i.e., straight arrays) may yield higher musical SQ. The straight electrode recipients had greater musical experience than the other participants in this sample (see below) which is a likely explanation; however, further work is needed to better understand this preliminary finding. A potential limitation of the current study was that many electrode conditions did not use the most basal or apical contact resulting in a downshift or upshift in frequencies compared to the participants’ clinical maps. Changing the number of electrodes invariably changed the spiral ganglion cells being stimulated in response to different frequencies, which could have potentially had an effect on music perception and subjective musical SQ ratings. Further, the strong positive correlation between greater variance in $\bar{M}$ for ST pre-curved electrodes, but not for ST straight electrodes also supports the idea that greater electrode-to-modiolus distance is advantageous for musical SQ. Of note, the straight electrode recipients did have greater musical experience than the other participants in this sample (see below), which is a likely contributor; however, further work is needed to better understand these preliminary findings.

Another related consideration is that the straight and pre-curved electrode recipients may have had different levels of underlying neural health which could impact perceptual quality. Specifically, it is quite possible that participants with straight electrodes were those with better preoperative hearing given that acoustic hearing preservation rates are generally higher for straight electrode arrays. We did not complete any measurements thought to reflect underlying neural health, such as multipulse integration (Zhou and Dong, 2017; Zhou et al., 2018), polarity sensitivity (e.g., Macherey et al., 2017; Hughes et al., 2018; Jahn and Arenberg, 2019), or amplitude growth functions for electrically evoked compound action potentials (e.g., Schvartz-Leyzac and Pfingst, 2016; He et al., 2018; Hughes et al., 2018). We did, however, complete two-tailed *t*-tests comparing preoperative audiometric thresholds for the CI ear in the straight and pre-curved electrode groups for LFPTA (125, 250, and 500 Hz), traditional PTA (500, 1000, and 2000 Hz), and high-frequency PTA (2000, 4000, and 8000 Hz). These analyses revealed no significant differences between preoperative audiometric thresholds in the CI ear across groups for LFPTA (*t*_19_ = −0.504, *p* = 0.62), PTA (*t*_19_ = 0.255, *p* = 0.80), or HFPTA (*t*_19_ = 2.07, *p* = 0.053). Nevertheless, we recognize that audiometric thresholds cannot necessarily serve as a surrogate for underlying neural health and as such, additional investigation into this relationship is warranted. Furthermore, given that our recipient recruitment did not control for musical experience nor was electrode group assignment completed randomly, we believe that the relationship between electrode array type and overall music ratings is confounded by both musical experience as well as the potential for preoperative device selection bias. We plan to investigate this relationship further in future investigations.

Another potential limitation of this study may be that the current measures are not sensitive enough to accurately measure spectral resolution because these results potentially suggest that greater channel interaction may be related to higher musical SQ ratings. Future studies should investigate more direct measures of spectral resolution, such as using the Quick Spectral Modulation Detection (QSMD) task (Gifford et al., 2014). Furthermore, many electrode conditions did not use the most basal or apical contact resulting in a downshift or upshift in frequencies compared to the participants’ clinical maps when the frequency tables were reallocated. Changing the number of electrodes invariably changed the spiral ganglion cells being stimulated in response to different frequencies, which could have potentially had an effect on music perception and subjective musical SQ ratings.

Hypothesis 2: *Participants with more musical experience, better frequency discrimination ability, and longer device use would rate musical SQ higher than participants with less experience and ability.*

As described above, participants with straight electrodes rated musical SQ to be significantly higher than participants with pre-curved arrays. Rather than concluding that greater $\bar{M}$ values are more desirable for musical SQ, we believe that patient factors may explain this result. Specifically, our sample of straight electrode recipients had significantly greater musical experience, as measured by the Ollen Index of Musical Sophistication, than our sample of pre-curved electrode recipients. This was driven primarily by S16 and S21, who were both serious amateur musicians. These two subjects also had more pre-operative hearing than traditional CI candidates which likely influenced surgeon selection of a straight electrode array. In their everyday settings, both subjects use EAS stimulation, which could influence their perception and appreciation of music differently than CI recipients who use electric stimulation for the full bandwidth (though the acoustic earhook was not used for the current study). After removing data from these two musicians, the positive correlation between musical experience and overall musical SQ ratings is greatly reduced and becomes non-significant.

These results contraindicated our hypothesis that participants with more musical experience would demonstrate higher overall musical SQ ratings. In the broader data set, there was no relationship found between pre-operative hearing thresholds and overall musical SQ and there was no difference in pre-operative hearing thresholds between electrode types. Using behavioral hearing thresholds as a correlate for greater neural survival, this suggests that greater neural survival did not significantly influence musical SQ ratings. However, this finding is very preliminary and the impact of neural survival on musical SQ should be investigated further in future studies.

While the Ollen scores of straight electrodes were significantly and positively correlated with overall musical SQ ratings, there was no significant relationship between these two measures for pre-curved electrode recipients. Again, when the data for the two musicians in our sample were removed, the positive correlation between straight electrode recipient Ollen scores and overall musical SQ ratings is greatly reduced and also becomes non-significant. The non-significant relationship between music perceptual accuracy and perceived musical SQ ratings found in this study is in keeping with previous studies (Gfeller et al., 2008; Looi et al., 2011) and suggests that music appreciation and SQ ratings cannot be predicted by measuring music perception abilities. Currently, music perception is rarely measured in the clinic, but measures of musical SQ are almost never included in CI clinical protocols. This is hugely problematic because CI recipients consistently report musical SQ impairments following implantation, and musical SQ is rated as the most significant factor responsible for music listening enjoyment (Lassaletta et al., 2008a, b; Roy et al., 2012). Future work should consider clinically feasible measures of musical SQ to address this gap in our battery of clinical assessments.

Although CI surgeons have traditionally selected straight electrodes for patients with greater pre-operative audiometric thresholds due to their lower rates of translocation compared to pre-curved arrays (e.g., O’Connell et al., 2016), this was not the case for our sample. Our results indicated no difference in pre-operative standard and LFPTAs between recipients with straight electrodes and those with pre-curved arrays. We also did not see an effect of surgeon, suggesting that surgical technique did not impact musical SQ ratings for this sample. Although it was not the aim of the current study, it is possible that the straight electrode recipients in this sample had shorter durations of deafness, better neural survival, or some combination of these factors compared to the participants with pre-curved electrodes. Future studies should more rigorously assess these factors as they relate to musical SQ and channel independence.

Hypothesis 3: *CI recipients with higher speech recognition performance and speech SQ ratings will rate musical SQ higher than poorer performing CI recipients.*

Speech recognition and speech SQ tasks were found to be significantly and positively correlated with musical SQ ratings. These positive relationships between speech recognition, speech SQ, and musical SQ may be useful for managing realistic expectations for patients in the Audiology clinic. Those patients who perform better on speech recognition tasks may also experience better perceptual SQ for speech and music stimuli. While previous literature has found a positive relationship between speech recognition performance and music perception abilities (Gfeller et al., 2003), the relationship between speech recognition and musical SQ, as well as speech SQ and musical SQ have not been explored prior to the current study. These relationships should continue to be explored in future studies to better individualize expectations management for music appreciation with a CI, as well as to potentially develop music-based intervention. This positive relationship between speech recognition and musical SQ also emphasizes the importance of including a measure of musical SQ in the clinical test battery, even for patients who do not consider music appreciation of high importance in their quality of life.

### Musical Genre Effects

All musical genres examined in the current study demonstrated significant increases in musical SQ ratings beyond four channels except for Hip Hop and Rap, perhaps due to the emphasis on rhythmic features and spoken lyrics often present in this genre. However, there was considerable variability both within and across genres for this relatively small population. While previous research has looked at CI recipients’ preference for less complex genres of music (Gfeller et al., 2003), no published studies have specifically investigated musical SQ ratings of various musical genres in an acute setting or as a function of the number of channels. Even though participants were instructed not to include music familiarity or preference in the selection of SQ ratings, it was not possible to eliminate the potential for participant bias in the present study. This potential bias should be considered in future studies, perhaps by limiting all samples to original/unfamiliar music or individualized music selections.

## Summary

The findings of this study are summarized as follows:

• Musical SQ ratings significantly increased from 4 to 10 independent channels.
  ○ Regression slope coefficient for music SQ versus number of channels was significantly different from zero for pre-curved electrode recipients, but not for straight electrode nor translocated electrode recipients.

• Musical SQ ratings were significantly higher for adult CI recipients with:
  ○ Electrodes localized to ST compared to those with translocated arrays.
  ○ Pre-curved electrodes that had more variability in electrode-to-modiolus distance across the array.
• Musical experience was not correlated with higher musical SQ ratings after controlling for two participants with significantly greater musical experience than the rest of the sample.
  ○ Control of patient variables including musical experience and music selection familiarity is recommended for future studies.
• There was no relationship between CI experience and musical SQ ratings.
• Musical SQ ratings were significantly and positively correlated with both speech recognition scores, in RAU, and speech SQ ratings.
• Musical SQ ratings significantly increased beyond four channels for all genres except Hip Hop and Rap.
  ○ There was considerable within- and between-genre variability in SQ ratings.
  ○ Future investigation into genre effects may prove useful for identifying recommended music listening progression for auditory training of newly implanted patients.

## Data Availability Statement

The datasets generated for this study are available on request to the corresponding author.

## Ethics Statement

The studies involving human participants were reviewed and approved by Vanderbilt University Institutional Review Board. The patients/participants provided their written informed consent to participate in this study.

## Author Contributions

KB and RG contributed to the conception and design of the study. JN, BD, RL, and RD contributed to collect the imaging data and perform the imaging analysis. KB recruited the potential subjects and conducted the experiments, and wrote the first draft of the manuscript with significant input from RG, RD, and VR. VR helped with the data analysis. All authors contributed to the manuscript revisions, and read and approved the submitted version of the manuscript.

## Conflict of Interest

The authors declare that the research was conducted in the absence of any commercial or financial relationships that could be construed as a potential conflict of interest.

## Footnotes

1 http://angelsound.tigerspeech.com/
